# Hepatitis B Virus Incidence and Risk Factors Among Human Immunodeficiency Virus-1 Negative Men Who Have Sex With Men in Kenya

**DOI:** 10.1093/ofid/ofw253

**Published:** 2016-12-07

**Authors:** Elizabeth Wahome, Caroline Ngetsa, John Mwambi, Huub C. Gelderblom, Gloria Omosa Manyonyi, Murugi Micheni, Amin Hassan, Matt A. Price, Susan M. Graham, Eduard J. Sanders

**Affiliations:** 1 KEMRI-Wellcome Trust Research Programme Centre for Geographic Medicine Research–Coast, Kilifi, Kenya; 2 International AIDS Vaccine Initiative, New York, New York; 3 KAVI Institute of Clinical Research, University of Nairobi, Kenya; 4 College of Health Sciences, School of Medicine, University of Nairobi, Kenya; 5 University of California at San Francisco, Department of Epidemiology and Biostatistics; 6 University of Washington, Seattle; 7 Nuffield Department of Medicine, University of Oxford, Headington, United Kingdom

**Keywords:** circumcision, HBV incidence, HIV-1 acquisition, Kenya, MSM

## Abstract

No data exist on hepatitis B virus (HBV) incidence among African men who have sex with men (MSM). We tested plasma samples archived between 2005 and 2014 for HBV core antibody or surface antigen seroconversion in a cohort of 312 initially human immunodeficiency virus (HIV)-1-negative MSM with no evidence of prior HBV infection. Hepatitis B virus incidence was 6.0/100 person-years (95% confidence interval [CI], 3.9–9.1). Hepatitis B virus acquisition was associated with being uncircumcised (adjusted incidence rate ratio [aIRR], 5.0; 95% CI, 1.5–16.8), recent HIV-1 acquisition (aIRR, 2.9; 95% CI, 1.1–7.7), rape (aIRR, 5.0; 95% CI, 1.2–20.4), and any tertiary education (aIRR, 3.2; 95% CI, 1.1–9.7). African MSM have a substantial risk of HBV acquisition and require vaccination urgently.

Chronic hepatitis B virus (HBV) infection, defined as persistence of HBV surface antigen (HBsAg) for greater than 6 months, is a leading cause of chronic liver disease and death worldwide [1]. In sub-Saharan Africa (SSA), the predominant mode of HBV transmission is horizontal, with a substantial number of infections acquired in early childhood [2, 3]. The estimated prevalence of chronic HBV infection in Kenya is 5.16% (95% confidence interval [CI], 4.86–5.48) [4]. Twenty-five percent of human immunodeficiency virus (HIV)-1-uninfected individuals with chronic HBV are estimated to develop cirrhosis, hepatocellular carcinoma (HCC), or both [1]. Approximately 40% of individuals who develop HBV-induced HCC in SSA do so before age 40 [5]. Although universal childhood HBV vaccination has been provided in Kenya since the early 2000s, adult male sex workers and other men who have sex with men (MSM) are not routinely offered HBV vaccination.

Human immunodeficiency virus-1 incidence among MSM in Kenya has been estimated at 8–10 per 100 person years [6, 7], with estimates as high as 35.2 (95% CI, 23.8–52.1) per 100 person years among MSM only [7]. Because HBV is more infectious than HIV-1 [8], it may be expected that HBV incidence is substantial among Kenyan MSM. However, no data exist on HBV incidence in this group, despite the need for such data to support HBV vaccination programming targeting MSM [9]. Therefore, we studied HBV incidence and associated risk factors for acquisition among initially HIV-1-negative, HBV-susceptible MSM enrolled in a cohort study in Coastal Kenya, all of whom were born before routine childhood vaccination began in Kenya.

## METHODS

### Study Design, Setting, and Population

We retrospectively tested stored plasma samples from HIV-1-negative MSM aged 18–49 years who were enrolled into a HIV-1 vaccine feasibility cohort study between 2005 and 2014 in Coastal Kenya [7]. All male cohort participants who reported anal sex with another man in the 3 months before enrollment and had stored blood samples available from enrollment and at least 1 follow-up visit through December 2014 were selected for the present study.

### Cohort Procedures

Detailed cohort procedures are described elsewhere [7]. In brief, enrollment and follow-up included a face-to-face interview using a standardized behavioral questionnaire, HIV-1 testing and counseling, medical history, and physical examination at each visit. Follow-up was quarterly, or monthly when receptive anal intercourse (RAI) was reported. Men who acquired HIV-1 during follow-up were offered enrollment into an acute infection cohort with quarterly follow-up visits during the first 2 years and 6-monthly visits thereafter [10]. From May 25, 2009 onwards, HBV vaccination was provided to cohort participants as part of ongoing care and prevention services. Follow-up visits after the first documented HBV vaccination date were excluded from the study.

### Laboratory Methods

We tested archived samples using 3 HBV serological assays: Murex HBsAg version 3 (Diasorin) for HBsAg, ETI-AB-AUK-3 (Diasorin) for hepatitis B surface antibody (anti-HBs), and Murex anti-HBc (Diasorin) for hepatitis B core antibody (anti-HBc). We first tested the last available follow-up sample. If this last sample was negative for all 3 markers, we did no further testing for that participant, assuming they would have negative serologies at baseline. If the last available sample was positive for any of the 3 HBV markers, we also tested the baseline sample. Any available in-between samples were tested for anti-HBc or HBsAg if the baseline sample was negative for all 3 markers and the last available sample was positive for anti-HBc or HBsAg.

Definitions used for this study were as follows: (1) HBV-susceptible, negative for all the 3 HBV markers at baseline or during follow-up; (2) resolved HBV-infection, positive for anti-HBc, with or without anti-HBs, but negative for HBsAg at baseline; (3) acute or chronic HBV-infection, positive for HBsAg at baseline; (4) incident HBV-infection, negative for all 3 HBV markers at baseline and positive for anti-HBc or HBsAg during follow-up, following the approach of Falade et al [11]); (5) possibly HBV-vaccinated, anti-HBs positive and negative for anti-HBc and HBsAg at baseline or during follow-up. The estimated date of infection for HBV was defined as the mid-point between a previously negative and subsequently positive anti HBc or HBsAg result [8, 11].

Detailed HIV-1 testing procedures are described elsewhere [7]. In brief, HIV-1 testing was performed at each study visit using 2 rapid antibody test kits (Determine, Abbott Laboratories; Unigold, Trinity Biotech) in parallel. Discordant rapid HIV-1 test results were resolved using an enzyme-linked immunohsorbent assay (ELISA) test (Genetic System HIV-1/2 plus O EIA; Bio-Rad). All HIV-1-negative samples were tested for p24 antigen (Vironostika HIV-1 p24 ELISA; Biomérieux), and pre- and post-seroconversion samples were tested for HIV-1 ribonucleic acid (Amplicor Monitor 1.5; Roche).

### Data Analysis and Statistical Methods

We compared demographic and behavioral characteristics of men who returned for follow-up to those who did not return for follow-up after enrollment using Pearson’s χ^2^ test. Hepatitis B virus-susceptible MSM were compared with MSM who had resolved, acute, or chronic HBV infection at baseline using Pearson’s χ^2^ test, after excluding men whose serology results indicated possible HBV vaccination. Prevalence ratios were used to measure associations between participant characteristics and resolved, acute, or chronic HBV infection. We included factors associated with resolved, acute, or chronic HBV infection at *P* less than 0.2 in the bivariate analysis in a multivariable Poisson model to estimate adjusted prevalence ratios.

We included MSM who were HBV-susceptible at enrollment in the incidence analysis. The primary outcome was incident HBV infection, calculated as the number of seroconversions to a positive anti-HBc or HBsAg result divided by person-years of follow-up in each group of interest. Individual follow-up time was calculated from the date of study entry until the estimated date of incident HBV infection, the date of first documented HBV vaccine dose, or the date prior to an isolated positive anti-HBs result (i.e., possible HBV vaccination). We censored participants who were not vaccinated or became lost to follow-up at the last visit date in the study for which a stored sample was available for testing.

We evaluated potential risk factors that were fixed (demographics, baseline circumcision status) or time-dependent (genital washing, alcohol use, intravenous drug use, sexual behaviors, HIV status). Poisson models with robust standard errors were used to obtain population-averaged incidence rate ratios that accounted for correlation due to repeated measurements on the same subject over time. We included variables significant in bivariable analysis at *P* < .2 and a priori predictors (associated with resolved, acute, or chronic HBV infection at enrollment in the univariate analysis) including age, marital status, sex work, RAI, and any unprotected sex, in an initial multivariable model of potential predictors of HBV acquisition. We then dropped variables with *P* > .1 in this initial model that were not a priori predictors, to produce the final multivariable model. *P* values were 2-sided and significance was set at *P* ≤ .05. We used Stata 13.0 (StataCorp LP, College Station, TX) for analysis.

### Ethical Considerations

The Kenya Medical Research Institute Ethics Review Committee approved the study. All participants provided written informed consent.

## RESULTS

### Study Population

Of 908 MSM screened for cohort enrollment between 2005 and 2014, 133 (14.6%) were HIV-1 positive and excluded. Of the 775 HIV-1-negative men enrolled, 198 (25.5%) did not return for any follow-up.

Compared to MSM who remained in follow-up, MSM who were lost to follow up after the enrollment visit were younger (60.1% vs 45.1% were 18–24 years of age, *P* = .001), less likely to have been married (89.4% vs 79.7% were never married, *P* = .002), and more likely to report insertive anal intercourse (IAI) (68.2% vs 58.8%, *P* = .019) (data not shown).

**Figure 1.**
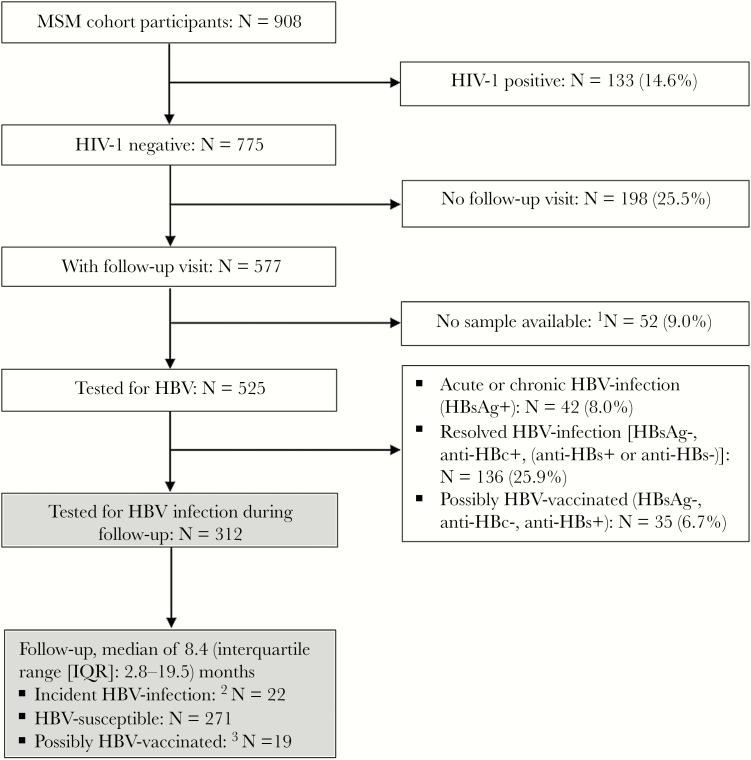
Flow chart of men who have sex with men (MSM) cohort participants selected for hepatitis B virus (HBV) acquisition study, Kilifi, Kenya, 2005–2014. ^1^Missing either enrollment or follow-up sample. ^2^Defined as negative for HBV surface antigen (HBsAg), hepatitis B surface antibody (anti-HBs), and hepatitis B core antibody (anti-HBc) at baseline and positive for anti-HBc or HBsAg at any point during follow-up, regardless of whether exposure resulted in persistent HBV infection. ^3^Anti-HBs conversion without evidence of HBV exposure, possibly vaccinated outside our research cohort.

Out of 577 HIV-1-negative MSM with at least 1 follow-up visit, 525 (91%) had enrollment and follow-up samples available for hepatitis B serology testing. Of these 525 MSM, 136 (25.9%) were classified as having resolved HBV infection, 42 (8.0%) as having acute or chronic HBV infection, and 35 (6.7%) as having possibly been vaccinated, because their serology was positive for anti-HBs only. A total of 312 (59.4%) were HBV susceptible at baseline (details for all participants in Figure 1).

### Hepatitis B Virus Prevalence and Risk Factors

Table 1 compares baseline demographic and behavioral characteristics of 312 HBV-susceptible and 178 MSM with resolved, acute, or chronic HBV-infections. Younger age, having never been married, sex work, RAI, and any unprotected sex were associated with HBV susceptibility. In multivariable analysis, only age remained statistically significantly associated with resolved, chronic, or acute HBV infection status, with younger men more likely to be HBV-susceptible (Supplemental Material, Table 1).

**Table 1.** Baseline Demographic and Behavioral Characteristics of 490 HIV-1 Negative MSM in Kilifi, Kenya, 2005–2014

Characteristics	Overall (n = 490)		HBV-Susceptible* (n = 312)		Resolved, Acute, or Chronic HBV Infection* (n = 178)		*P* Value^†^
	n	(%)	n	(%)	n	(%)	
Age group (years)							
18–24	254	(51.8)	178	(57.1)	76	(42.7)	.001
25–34	188	(38.4)	113	(36.2)	75	(42.1)	
35+	48	(9.8)	21	(6.7)	27	(15.2)	
Education							
Primary/none	249	(50.8)	149	(47.8)	100	(56.2)	.193
Secondary	191	(39.0)	130	(41.7)	61	(34.3)	
Higher/tertiary	50	(10.2)	33	(10.6)	17	(9.6)	
Marital status							
Never married	405	(82.7)	267	(85.6)	138	(77.5)	.024
Ever married	85	(17.3)	45	(14.4)	40	(22.5)	
Religion							
Christian	234	(47.8)	156	(50.0)	78	(43.8)	.405
Muslim	146	(29.8)	90	(28.8)	56	(31.5)	
Other/none	110	(22.4)	66	(21.2)	44	(24.7)	
Employment							
None	203	(41.4)	135	(43.3)	68	(38.2)	.504
Self	218	(44.5)	133	(42.6)	85	(47.8)	
Formal	69	(14.1)	44	(14.1)	25	(14.0)	
Circumcised							
Yes	456	(93.1)	294	(94.2)	162	(91.0)	.177
Sex partners past 3 months							
Men and women	306	(62.4)	190	(60.9)	116	(65.2)	.348
Men only	184	(37.6)	122	(39.1)	62	(34.8)	
Received payment for sex with cash, living expenses, or goods in past 3 months							
Yes	337	(68.8)	225	(72.1)	112	(62.9)	.035
Paid for sex in past 3 months							
Yes	157	(32.0)	98	(31.4)	59	(33.1)	.692
Any use of alcoholic beverages in past month							
Yes	320	(65.3)	206	(66.0)	114	(64.0)	.658
Receptive anal intercourse (RAI) in past 3 months							
Yes	303	(61.8)	203	(65.1)	100	(56.2)	.052
Insertive anal intercourse (IAI) past in 3 months							
Yes	288	(58.8)	183	(58.7)	105	(59.0)	.942
Sexual exposure and protection with condom in past week							
No activity	99	(20.2)	61	(19.6)	38	(21.3)	.012
All protected	90	(18.4)	46	(14.7)	44	(24.7)	
Any unprotected	301	(61.4)	205	(65.7)	96	(53.9)	
Total sex partners in past month							
Less than 3	168	(34.3)	101	(32.4)	67	(37.6)	.237
Three or more	322	(65.7)	211	(67.6)	111	(62.4)	
Group sex in past 3 month							
Yes	83	(16.9)	52	(16.7)	31	(17.4)	.832
Been raped in past 3 month							
Yes	11	(2.2)	6	(1.9)	5	(2.8)	.524
Intravenous drug use in past 3 months							
Yes	8	(1.6)	4	(1.3)	4	(2.2)	.417
Genital washing with soap in past week							
Yes	399	(81.4)	252	(80.8)	147	(82.6)	.619
Sex with a menstruating female partner in past 3 months							
Yes	51	(10.4)	35	(11.2)	16	(9.0)	.490
Don’t know	2	(0.4)	2	(0.6)	0	(0.0)	

Abbreviations: anti-HBc, hepatitis B core antibody; HBsAg, hepatitis B virus surface antigen; anti-HBs, hepatitis B surface antibody; HBV, hepatitis B virus; HIV, human immunodeficiency virus; MSM, men who have sex with men.

*HBV-susceptible was defined as negative for all 3 HBV markers studied (HBsAg, anti-HBs, anti-HBc); resolved HBV infection was defined as positive for anti-HBc (with or without anti-HBs); and acute or chronic HBV-infection was defined as positive for HBsAg. We excluded participants who were positive for HBV surface antibody only, who were considered to have possibly received HBV vaccine.

^†^Two-sided value based on χ^2^ test for proportions.

### Hepatitis B Virus Incidence and Risk Factors

Overall, we followed 312 MSM for a median of 8.4 months (interquartile range, 2.8–19.5) contributing 369.0 person-years. Twenty-two MSM converted for anti-HBc or HBsAg during follow-up, giving a crude HBV incidence rate of 6.0 (95% CI, 3.9–9.1) per 100 person-years. Of these, 5 (22.7%) acquired HIV-1 at a median of 30 months (range, 13–36 months) before acquiring HBV infection, whereas the remaining 17 (77.3%) acquired HBV while remaining HIV-1 seronegative. Of 27 men who acquired HIV-1 during follow-up, 4 started lamivudine or tenofovir-containing antiretroviral therapy (ART) during follow-up, at 24 to 55 months after HIV-1 acquisition. One of these 4 MSM converted for anti-HBc, at ~12 months after ART initiation.

In the multivariable analysis adjusted for age, marital status, sex work, RAI, and any unprotected sex, uncircumcised status (adjusted incidence rate ratio [aIRR], 5.0; 95% CI 1.5–16.8), recent HIV-1 acquisition (aIRR, 2.9; 95% CI 1.1–7.7), rape (5.0; 95% CI 1.2–20.4) and any tertiary education (aIRR, 3.2; 95% CI 1.1–9.7) were associated with HBV acquisition (Table 2). Results did not change when using a time-updated circumcision variable to capture the status of 1 man who was circumcised during follow-up (and remained HBV susceptible at his last visit). Of note, the unadjusted association between uncircumcised status and HBV infection was stronger among MSM reporting IAI (incidence rate ratio [IRR], 4.6; 95% CI 1.0–20.4) than among men not reporting IAI (IRR, 3.5; 95% CI 0.9–14.3). The 8 incident HBV cases among men reporting RAI all occurred in circumcised MSM.

**Table 2.** Risk Factors Associated With HBV Incidence Among 312 MSM in Kilifi, Kenya, 2005–2014

Characteristics	Incident HBV/100 PY*	Incidence/100 PY (95% CI)	Unadjusted IRR (95% CI)	*P* Value	Adjusted IRR (95% CI)	*P* Value
Age categories (years)†						
18–24	12/204.4	5.9 (3.3-10.3)	Reference		Reference	
25–34	9/133.4	6.7 (3.5-13.0)	1.15 (0.48-2.75)	.755	1.09 (0.46-2.57)	.840
35+	1/31.3	3.2 (0.4–22.7)	0.54 (0.07-4.33)	.564	0.74 (0.11-4.89)	.752
Education						
Primary/none	9/185.0	4.9 (2.5-9.4)	Reference		Reference	
Secondary	9/160.9	5.6 (2.9-10.8)	1.15 (0.46-2.92)	.762	1.00 (0.41-2.45)	.998
Higher/tertiary	4/23.2	17.2 (6.5-45.9)	3.56 (1.15-11.00)	.027	3.18 (1.05-9.65)	.041
Marital status†						
Never married	20/303.3	6.6 (4.3-10.2)	Reference		Reference	
Ever married	2/65.7	3.0 (0.8–12.2)	0.46 (0.10-2.02)	.303	0.51 (0.12-2.08)	.346
Religion						
None/other	3/51.0	5.9 (1.9-18.2)	Reference		–	–
Christian	14/184.9	7.6 (4.5-12.8)	1.29 (0.36-4.66)	.700		
Muslim	5/133.1	3.8 (1.6-9.0)	0.64 (0.14-2.80)	.549		
Employment						
None	11/165.1	6.7 (3.7-12.0)	Reference		–	–
Self	8/138.6	5.8 (2.9-11.5)	0.87 (0.35-2.16)	.764		
Formal	3/65.3	4.6 (1.5-14.2)	0.69 (0.18-2.57)	.578		
Circumcised						
Yes	18/349.4	5.2 (3.2-8.2)	Reference		Reference	
No	4/19.6	20.4 (7.7-54.3)	4.00 (1.44-11.08)	.008	4.96 (1.47-16.75)	.010
HIV-1 status						
Seronegative	17/325.2	5.2 (3.2-8.4)	Reference		Reference	
Recent HIV acquisition (range, 13–36 months)	5/43.8	11.4 (4.8-27.4)	2.19 (0.83-5.74)	.111	2.88 (1.08-7.67)	.035
Sex partners past 3 months						
Men and women	15/252.2	5.9 (3.6-9.9)	Reference		–	–
Men only	7/116.8	6.0 (2.9-12.6)	1.01 (0.41-2.48)	.989		
Received payment for sex with cash, living expenses, or goods in past 3 months^†^						
No	11/159.6	6.9 (3.8-12.4)	Reference		Reference	
Yes	11/209.4	5.3 (2.9-9.5)	0.76 (0.32-2.81)	.538	0.75 (0.31-1.85)	.537
Paid for sex in past 3 months						
No	16/261.3	6.1 (3.8-10.0)	Reference		–	–
Yes	6/107.7	5.6 (2.5-12.4)	0.91 (0.35-2.35)	.846		
Any use of alcoholic beverage in past month						
No	10/139.4	7.2 (3.9-13.3)	Reference		–	–
Yes	12/229.6	5.2 (3.0-9.2)	0.73 (0.31-1.73)	.475		
Receptive anal intercourse (RAI) in past 3 months^†^						
No	14/203.8	6.9 (4.1-11.6)	Reference		Reference	
Yes	8/165.2	4.8 (2.4-9.7)	0.70 (0.30-1.67)	.426	0.46 (0.19-1.13)	.091
Insertive anal intercourse (IAI) in past 3 months^‡^						
No	12/180.5	6.6 (3.8-11.7)	Reference		–	–
Yes	10/188.5	5.3 (2.9-9.9)	0.80 (0.34-1.86)	.601		
Sexual exposure and protection with condoms in past week^†^						
No activity	6/120.3	5.0 (2.2-11.1)	Reference		Reference	
All protected	6/108.7	5.5 (2.5-12.3)	1.11 (0.36-3.43)	.859	1.42 (0.45-4.53)	.549
Any unprotected	10/140.0	7.1 (3.8-13.3)	1.44 (0.51-4.00)	.490	2.06 (0.67-6.34)	.208
Total sex partners in past month						
Less than 3	11/192.4	5.7 (3.2-10.3)	Reference		–	–
Three or more	11/176.6	6.2 (3.4-11.2)	1.09 (0.48-2.48)	.838		
Group sex in past 3 months^‡^						
No	18/333.2	5.4 (3.4-8.6)	Reference			
Yes	4/35.9	11.2 (4.2-29.7)	2.06 (0.69-6.15)	.196		
Raped in past 3 months						
No	21/364.5	5.8 (3.8-8.8)	Reference		Reference	
Yes	1/4.5	22.0 (3.1–156.2)	3.82 (0.92-15.80)	.064	4.95 (1.20-20.42)	.027
Intravenous drug use in past 3 months						
No	22/368.0	6.0 (3.9-9.1)	–	–	–	–
Yes	0/1.0	0				
Genital washing with soap in past week^‡^						
No	7/71.2	9.8 (4.7-20.6)	Reference		–	–
Yes	15/297.8	5.0 (3.0-8.4)	0.51 (0.21-1.25)	.140		
Sex with a menstruating female partner in past 3 months						
No	20/341.0	5.9 (3.8-9.1)	Reference		–	–
Yes	1/24.9	4.0 (0.6–28.5)	0.68 (0.09-5.01)	.701		
Don’t know	1/3.1	32.2 (4.5-228.7)	5.49 (0.67-44.96)	.113		

Abbreviations: aIRR, adjusted incidence rate ratios; CI, confidence interval; anti-HBc, hepatitis B core antibody; HBsAg, hepatitis B virus surface antigen; anti-HBs, hepatitis B surface antibody; HBV, hepatitis B virus; HIV-1, human immunodeficiency virus; IRR, incidence rate ratios; MSM, men who have sex with men; PY, person-years.

*Incident HBV infection was defined as being negative for HBsAg, anti-HBs, and anti-HBc at baseline and positive for anti-HBc or HBsAg at any point during follow-up, regardless of whether exposure resulted in persistent HBV infection.

^†^Considered a priori in the initial multivariable model.

^‡^Not included in the final model.

## DISCUSSION

In this population of MSM in coastal Kenya, approximately 60% were susceptible to HBV infection at enrollment. Susceptible men were young and reported high-risk sexual behavior that put them at risk for both HIV-1 and HBV acquisition. We found an overall incidence of HBV core or HBsAg seroconversion of 6.0 per 100 person-years. Recent HIV-1 acquisition, having been raped and any tertiary education were strongly associated with HBV acquisition risk, whereas circumcised MSM had lower HBV acquisition rates.

A high HBV incidence was observed in our cohort among MSM compared with that reported in the Multicenter AIDS Cohort Study (MACS) cohort in the United States (~0.96 per 100 person-years) [11] and in Amsterdam (~1.0–2.0 per 100 person-years) [8]. Of note, 31% of men in the MACS study had received at least 1 dose of HBV vaccine, increasing to 60% by the end of the observation period. In our analysis, we excluded men positive for anti-HBs only at baseline and censored initially HBV-susceptible men at their first HBV vaccine dose, so our higher estimate reflects HBV incidence in an unvaccinated cohort.

The HBV acquisition risk in this study was 5-fold higher in those who were uncircumcised compared with circumcised MSM. To the best of our knowledge, our study is the first to report an association between HBV acquisition risk and lack of circumcision among MSM in Africa. A cross-sectional study conducted among MSM from Argentina showed no significant association between male circumcision and prior HBV infection, defined as a positive HBsAg or anti-HBc test, either overall or among MSM who reported no RAI [12]. Some studies of the association between circumcision status and HBV infection in other populations have reported an increased risk of HBV due to circumcision as one of several unsafe medical procedures or traditional practices examined [13]. It is possible that safe surgical removal of the foreskin reduces sexual HBV acquisition through similar mechanisms as for HIV-1 infection [14]. That no association has been found between circumcision and prevalent HBV infection in studies in SSA could be explained by the large number of HBV infections acquired during childhood [3] and the failure to evaluate HBV acquisition prospectively. Our finding that circumcision may protect against HBV acquisition needs verification in other populations with sexual HBV acquisition risk, including uncircumcised men who report IAI.

Men who have sex with men who had acquired HIV-1 in the past 1–3 years were 3-fold more likely to acquire HBV compared with men who remained HIV-1 negative during follow-up. This is consistent with a cohort study conducted among MSM in the United States that documented higher rates of incident HBV (defined as positive HBsAg or anti-HBc results during follow-up) in men who were HIV-infected at baseline, compared with men without HIV infection at baseline [11]. In that study, individuals who acquired HIV-1 during follow-up were reclassified as HIV-infected and not categorized separately as having recent HIV-1 acquisition. Although the strong association of HBV acquisition with recent HIV-1 acquisition in our study of initially HIV-1-negative men remained after adjusting for sexual risk behavior, it may be possible that men who acquired HIV-1 during follow-up were at higher risk for sexual HBV acquisition due to unmeasured confounders. In our study, most MSM who acquired HIV-1 were not immediately treated with ART because Kenyan guidelines during the study period required a CD4 count of 350 or below to initiate therapy. In future prospective studies in which HIV-1-positive men will be immediately treated, it is likely that HBV risk will be reduced [15].

We observed a 5-fold higher risk of HBV acquisition among men who were raped. Because there was only 1 event among men who reported rape, CIs for the risk estimate were wide; however, it is plausible that damage to mucosal surfaces during rape may enhance HBV acquisition.

The rate of HBV infection among men with any tertiary education was 3 times that of MSM with primary or no formal education. Of note, a lower proportion of MSM with tertiary education reported any unprotected sex compared to MSM with lower education (15.2% vs. 38.0%, *P* = .008) (data not shown). The association we report between HBV risk and tertiary education may be due to under-reporting of sexual risk behavior among MSM with tertiary education, or to unmeasured confounders.

This study had 3 main limitations. First, self-reported sexual behavior may have been subject to recall bias, and social desirability bias may have led to underreporting of risky sexual behavior resulting to reduced effect sizes. Second, our sample and the number of HBV acquisition events were small. This likely reduced power and affected our ability to test multiple associations. Third, the MSM we studied are not representative of all Kenyan MSM; they all reported high-risk sexual behavior and most participated in transactional sex. Thus, the HBV incidence we observed may overestimate HBV incidence among MSM in Kenya.

Universal childhood HBV vaccination was introduced in Kenya in 2002 [16]. However, MSM born before this date did not receive HBV vaccination during childhood and hence are susceptible to HBV infection later in life. They and other at-risk populations such as female sex workers and injection drug users should be targeted for HBV vaccination.

## CONCLUSIONS

In conclusion, we documented high HBV acquisition rates among MSM in Coastal Kenya, associated with recent HIV-1 acquisition, uncircumcised status rape, and tertiary education. Kenyan MSM who are at high risk for HIV-1 acquisition and for rape [17] should be prioritized for HBV vaccination. Given our findings on decreased HBV-infection risk in circumcised men, consideration should also be given to the promotion of circumcision among MSM who report IAI.

## Supplementary Data

Supplementary materials are available at *Open Forum Infectious Diseases* online. Consisting of data provided by the authors to benefit the reader, the posted materials are not copyedited and are the sole responsibility of the authors, so questions or comments should be addressed to the corresponding author.

## Supplementary Material

ofw253_suppl_supplemental_table_1
