# Supplementary material for: Hepatitis B Virus Incidence and Risk Factors Among Human Immunodeficiency Virus-1 Negative Men Who Have Sex With Men in Kenya
Source: Open Forum Infect Dis. 2016 Dec 7;4(1):ofw253. doi: 10.1093/ofid/ofw253 (PMC5499746; doi:10.1093/ofid/ofw253)
Supplement: ofw253_suppl_supplemental_table_1 [file ofw253_suppl_supplemental_table_1.docx]

| Supplemental Table 1. Factors associated with resolved, acute, or chronic HBV infection at enrollment among 490 MSM in Kilifi, Kenya 2005-2014 | | | | | | | |
| --- | --- | --- | --- | --- | --- | --- | --- |
|  | | **Resolved, acute, or chronic HBV infection*,** | **Univariate Analysis** | |  | **Multivariate Analysis** | |
| Characteristics | | **Proportion (%)** | **PR (95% CI)** | ***P* value** |  | **aPR (95% CI)** | ***P* value** |
| Age categories (years) | |  |  |  |  |  |  |
|  | 18-24 | 76/254 (29.9) | Reference |  |  | Reference |  |
|  | 25-34 | 75/188 (39.9) | 1.33 (1.03-1.72) | 0.029 |  | 1.29 (1.00-1.68) | 0.054 |
|  | 35+ | 27/48 (56.3) | 1.88 (1.37-2.57) | <0.001 |  | 1.65 (1.12-2.42) | 0.011 |
| Education | |  |  |  |  |  |  |
|  | Primary/none | 100/249 (40.2) | Reference |  |  | Reference |  |
|  | Secondary | 61/191 (31.9) | 0.80 (0.62-1.03) | 0.080 |  | 0.82 (0.63-1.06) | 0.124 |
|  | Higher/tertiary | 17/50 (34.0) | 0.85 (0.56-1.28) | 0.432 |  | 0.79 (0.53-1.19) | 0.255 |
| Marital status | |  |  |  |  |  |  |
|  | Never married | 138/405 (34.1) | Reference |  |  | Reference |  |
|  | Ever married | 40/85 (47.1) | 1.38 (1.06-1.80) | 0.016 |  | 1.01 (0.74-1.39) | 0.921 |
| Religion | |  |  |  |  |  |  |
|  | Christian | 44/110 (40.0) | Reference |  |  | - | - |
|  | Muslim | 78/234 (33.3) | 0.83 (0.62-1.12) | 0.221 |  |  |  |
|  | None/other | 56/146 (38.4) | 0.96 (0.70-1.30) | 0.789 |  |  |  |
| Employment | |  |  |  |  |  |  |
|  | None | 68/203 (33.5) | Reference |  |  | - | - |
|  | Self | 85/218 (39.0) | 1.16 (0.90-1.50) | 0.244 |  |  |  |
|  | Formal | 25/69 (36.2) | 1.08 (0.75-1.56) | 0.676 |  |  |  |
| Circumcised | |  |  |  |  |  |  |
|  | Yes | 162/456 (35.5) | Reference | 0.145 |  | Reference |  |
|  | No | 16/34 (47.1) | 1.32 (0.91-1.93) |  |  | 1.41 (0.95-2.09) | 0.087 |
| Sex partners past 3 months | |  |  |  |  |  |  |
|  | Men and women | 116/306 (37.9) | Reference |  |  |  |  |
|  | Men only | 62/184 (33.7) | 0.88 (0.69-1.14) | 0.353 |  |  |  |
| Received payment for sex with cash, living expenses, or goods in past 3 months | |  |  |  |  |  |  |
|  | No | 66/153 (43.1) | Reference |  |  | Reference |  |
|  | Yes | 112/337 (33.2) | 0.77 (0.61-0.98) | 0.031 |  | 0.81 (0.64-1.04) | 0.099 |
| Paid for sex in past 3 months | |  |  |  |  |  |  |
|  | No | 119/333 (35.7) | Reference |  |  | - | - |
|  | Yes | 59/157 (37.6) | 1.05 (0.82-1.35) | 0.691 |  |  |  |
| Any use of alcoholic beverage in past month | |  |  |  |  |  |  |
|  | No | 64/170 (37.6) | Reference |  |  | - | - |
|  | Yes | 114/320 (35.6) | 0.95 (0.74-1.21) | 0.657 |  |  |  |
| Receptive anal intercourse (RAI) in past 3 months | |  |  |  |  |  |  |
|  | No | 78/187 (41.7) | Reference |  |  | Reference |  |
|  | Yes | 100/303 (33.0) | 0.79 (0.63-1.00) | 0.049 |  | 0.87 (0.68-1.11) | 0.259 |
| Insertive anal intercourse (IAI) in past 3 months | |  |  |  |  |  |  |
|  | No | 73/202 (36.1) | Reference |  |  | - | - |
|  | Yes | 105/288 (36.5) | 1.01 (0.79-1.28) | 0.942 |  |  |  |
| Sexual exposure and protection with condoms in past week | |  |  |  |  |  |  |
|  | No activity | 38/99 (38.4) | Reference |  |  | Reference |  |
|  | All protected | 44/90 (48.9) | 1.27 (0.92-1.77) | 0.147 |  | 1.32 (0.95-1.84) | 0.083 |
|  | Any unprotected | 96/301 (31.9) | 0.83 (0.62-1.12) | 0.226 |  | 0.89 (0.65-1.20) | 0.443 |
| Total sex partners in past month | |  |  |  |  |  |  |
|  | Less than three | 67/168 (39.9) | Reference |  |  |  |  |
|  | Three or more | 111/322 (34.5) | 0.86 (0.68-1.10) | 0.473 |  |  |  |
| Group sex in past 3 months | |  |  |  |  |  |  |
|  | No | 147/407 (36.1) | Reference |  |  | - | - |
|  | Yes | 31/83 (37.3) | 1.03 (0.76-1.41) | 0.831 |  |  |  |
| Raped in past 3 months | |  |  |  |  |  |  |
|  | No | 173/479 (36.1) | Reference |  |  | - | - |
|  | Yes | 5/11 (45.5) | 1.26 (0.65-2.43) | 0.494 |  |  |  |
| Intravenous drug use in past 3 months | |  |  |  |  |  |  |
|  | No | 174/482 (36.1) | Reference |  |  | - | - |
|  | Yes | 4/8 (50.0) | 1.39 (0.69-2.80) | 0.364 |  |  |  |
| Genital washing with soap in past week | |  |  |  |  |  |  |
|  | No | 31/91 (34.1) | Reference |  |  | - | - |
|  | Yes | 147/399 (36.8) | 1.08 (0.79-1.48) | 0.625 |  |  |  |
| Sex with a menstruating female partner in past 3 months | |  |  |  |  |  |  |
|  | No | 162/437 (37.1) | Reference |  |  | - | - |
|  | Yes | 16/51 (31.4) | 0.85 (0.55-1.29) | 0.441 |  |  |  |
|  | Don’t know | 0/2 (0.0) | - |  |  |  |  |
| Abbreviations: HBV, Hepatitis B virus; CI, Confidence Interval; PR, prevalence ratios; aPR, adjusted prevalence ratios.  *HBV susceptible was defined as negative for all three HBV markers studied (HBsAg, anti-HBs, anti-HBc); resolved HBV infection was defined as positive for anti-HBc (with or without anti-HBs); and acute or chronic HBV infection was defined as positive for HBsAg.  We excluded participants who were positive for HBV surface antibody only, who were considered to have possibly received HBV vaccine. | | | | | | | |
